# The Challenge of Sexual Reproductive Health in Sierra Leone: Gendered Perceptions of Family Planning

**DOI:** 10.1002/puh2.70430

**Published:** 2026-11-06

**Authors:** Bea Arnfield, M. J. Le Vu, Sophie Ritzler, Constance Mulligan, Musa Santigie Kamara, Michael Kramp, Fathima Wakeel

**Affiliations:** ^1^ Lehigh University Bethlehem Pennsylvania USA; ^2^ Independent Community Health Worker (Unaffiliated) Makeni Sierra Leone

**Keywords:** family planning, gendered perspectives, health promotion, nonclinical intervention, qualitative research, self‐advocacy, sexual reproductive health, West Africa

## Abstract

**Background/Introduction:**

In Sierra Leone, persistent challenges in sexual and reproductive health (SRH), including high maternal mortality, poor maternal health outcomes, and gender inequality, underscore the critical importance of family planning. Despite progress, with contraceptive use among married women rising from 8% (2008) to 21% (2019), barriers related to knowledge and cultural norms remain. This study explored the family‐planning perceptions of mothers and fathers, using a short film as a discussion stimulus.

**Methods:**

We conducted 11 focus group discussions following the screening of a health‐promotion short film. Participants included 35 mothers and 21 fathers from the Bombali District. Discussions were analyzed using thematic analysis to identify gender‐based perspectives.

**Results:**

Mothers predominantly expressed positive views, emphasizing the benefits of family planning for self‐advocacy and control, child well‐being, and health. In contrast, fathers primarily focused on concerns about contraceptive side effects and the impact on partner relationships. A notable theme among fathers was criticism of providing family planning to adolescent girls, based on fears it would promote promiscuity.

**Conclusions:**

The stark contrast in perspectives underscores the need for family‐planning programs that are tailored to address gender‐specific concerns and that promote constructive communication amongst couples. The film effectively elicited nuanced discussions, suggesting that narrative‐based media are valuable tools for qualitative research and health promotion. These findings provide key insights for refining SRH interventions in Sierra Leone and similar contexts.

## Graphical abstract

Gendered norms shape family‐planning decisions in Sierra Leone: Fathers seek involvement yet reinforce stigma and control, whereas mothers emphasize health, stability, and education. Narrative film‐based interventions can reduce stigma, correct misinformation, and foster partner communication by supporting more equitable, informed reproductive decision‐making without undermining women's autonomy.

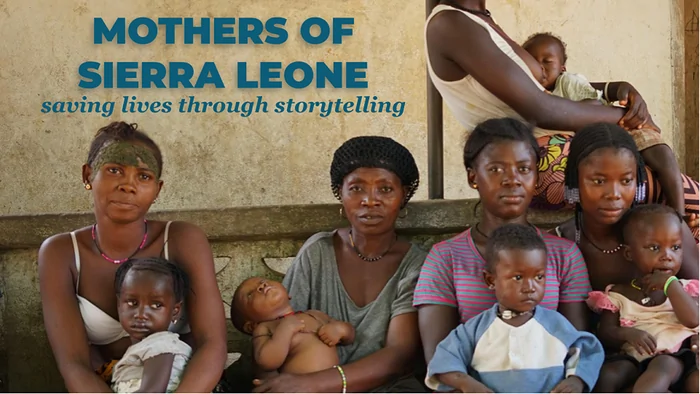

## 1 Introduction

Sexual and reproductive health (SRH) remains a pressing issue throughout Sierra Leone and sub‐Saharan Africa. SRH is one of the key determinants of a nation's high maternal mortality rates, high unplanned birth rates, and gender inequalities. Family planning is a vital aspect of SRH, empowering women to make choices about pregnancy and the overall health of their bodies. Family planning is defined as the information and practice of controlling the number and timing of children in a family, including the use of contraceptives or abstinence, as well as access to education on reproductive health [1]. The inadequate or incorrect use of family‐planning methods contributes to the adolescent pregnancy rate in Sierra Leone (28%), where adolescents account for 40% of all maternal deaths [2, 3]. This article will focus on differing perceptions between mothers and fathers about family‐planning methods, specifically child spacing and contraceptive use, and discuss the implications of these methods for SRH.

Family planning decreases the number of total and high‐risk births [4]. Previous initiatives, such as the Sierra Leone National Reproductive, Maternal, Newborn, Child, and Adolescent Health Strategy, have strengthened the access to and use of family‐planning resources. These initiatives have contributed to an increased rate of long‐acting reversible contraceptive (LARC) use [5]. LARC methods include intrauterine devices (IUDs), birth control implants, and birth control shots, colloquially referred to as the “captain ban,” the “coil,” and “depo” [6]. Contraceptive use increased among all women in Sierra Leone, from 8% in 2008 to 24% in 2019 [2]. In an effort to continue improving family‐planning resources, the Ministry of Health in Sierra Leone has announced its FP2030 commitments, including increasing access, improving policy, strengthening supply chains, establishing sustainable financing, and shifting social and gender norms, as a part of the global initiative to advance family planning [7].

### 1.1 Past Research

In Sierra Leone, family planning is a household‐level decision shaped by couples, families, and broader social norms. Women are and should be at the center of reproductive health decision‐making, yet their choices are influenced by other factors, such as partner approval, community expectations, access to accurate information, and prevailing beliefs about contraception [8]. Consequently, reproductive decision‐making is relational. Within this context, we use the term interagency to describe women's ability to navigate, negotiate, and collaboratively make family‐planning decisions within the interpersonal relationships and social structures that shape their reproductive choices. Unlike individual agency, which emphasizes autonomous decision‐making, interagency recognizes that reproductive decision‐making often occurs through interactions with partners, family members, and healthcare providers, particularly in settings where women's individual agency may be constrained. Strengthening family‐planning outcomes therefore requires supporting communication within couples and addressing the social conditions that shape reproductive choices.

Men in Sierra Leone report low involvement in women's SRH because of social and gender norms. A study of fathers in the Kono District of Sierra Leone found that men reported very low usage of male contraceptive options and mainly perceived family planning as a woman's concern [9]. Most commonly, fathers highlighted financial contributions to access family‐planning methods as the extent of their involvement. Fathers also mentioned negative perceptions of prevention methods, emphasizing unsafe side effects for women, including possible infertility [9]. This pattern is commonly found in Sierra Leone, as men and women consider accessing and using contraception options—along with other health concerns tied to pregnancy and birth—to be a woman's domain [10]. Although low involvement and negative perceptions of family planning are well‐documented in sub‐Saharan Africa, important gaps remain. Limited research has compared male perceptions of their involvement in family planning to female perceptions of the benefits of family planning for themselves, their families, and their communities. Appropriate men's involvement in SRH and family planning is essential to reduce stigma and ensure successful program development.

### 1.2 Current Study

In this study, the film [Blinded for Review] was shown and discussed in a series of focus groups. The focus group discussion (FGD) findings reveal differences in perceptions of family planning between mothers and fathers and inform future initiatives about improving family planning in Sierra Leone, especially those tailored to gender‐specific perceptions and improving women's interagency within the Sierra Leonean family structure.

### 1.3 Objective

This study seeks to address the following research questions: (1) How do mothers and fathers in Bombali District perceive their own roles and their partners’ roles in family‐planning decision‐making, and (2) how do perceptions of contraceptive benefits, side effects, and acceptability differ between mothers and fathers?

## 2 Methods

### 2.1 Sample and Procedures

Data are from seven focus groups with a total of 35 mothers at the World Hope International office, Makama Health Post, Holy Spirit Hospital, Makeni Regional Government Hospital, and Kunsho Community Health Centre, and four focus groups with a total of 21 fathers at the World Hope International office and the Child Health and Mortality Prevention Surveillance (CHAMPS) office. FGDs were organized by gender to create safe and culturally appropriate spaces that encouraged open expression of views, particularly in a context where gender norms may influence participation and disclosure. Separating mothers and fathers minimized power dynamics and social desirability bias that could arise in mixed‐gender settings. This design also strengthened the study's comparative objective by allowing independent exploration of maternal and paternal perceptions, thereby facilitating clearer cross‐gender analysis. This study was approved by the Lehigh University Institutional Review Board and Sierra Leone Ethics and Scientific Review Committee, Directorate of Training and Research.

Focus group participants were recruited by a community research partner via convenience sampling using WhatsApp community groups and participant referrals. Eligible participants were women and men of reproductive age. Individuals with impairments that could interfere with comprehension of the film were excluded from participation. Participants received light refreshments as compensation for their time. No monetary incentives were provided.

Verbal informed consent was obtained prior to beginning the focus group. Focus groups were conducted by our community research partners who are fluent in Krio and other local languages, such as Temne, ensuring adequate understanding for all focus group participants. The focus groups, including the provision of informed consent, were recorded, transcribed verbatim in Krio, and subsequently translated into English by bilingual members of the research team. Prior to participation, all participants provided informed consent and were informed that the FGDs would be audio‐recorded for research purposes. Participation in the recorded focus groups was voluntary.

### 2.2 Intervention

The film is a short documentary‐style educational video developed by [Blinded for Review], a project by [Blinded for Review] University to support community education on maternal health in Sierra Leone. The film combines interview‐based narration with observational footage and voice‐over commentary. It presents a frontline healthcare perspective, primarily through a registered nurse describing clinical practices and counseling procedures related to family planning and maternal health. The main contraceptive methods discussed in the film were condoms, combined oral contraceptive (COC) pills, depo injections, and IUDs or coils. The narrative is linear in structure, moving from general statements about maternal mortality to specific descriptions of contraceptive counseling, confidentiality practices, and protection efforts against pregnancy and sexually transmitted infections. The total runtime is approximately 3 min and 20 s, and the tone is informative and educational, consistent with public health training or awareness media. The film was pilot‐tested with two maternal health educators in the United States and Sierra Leone.

### 2.3 Measures

The film shown in focus groups featured the Kunsho Community Health Post (CHP), Bombali District, where a state‐registered nurse discussed family‐planning methods with an emphasis on the importance of child spacing and contraceptive options. Participants in the focus groups were asked about their opinions on the film's style, content, and the topic. The [Blinded for Review] team completed a practice focus group with healthcare workers and a community research partner in Sierra Leone before beginning the formal data collection. On the basis of feedback from these practice sessions, questions were modified, and probes were added to assist the translators in focus group facilitation.

### 2.4 Analysis

Thematic analysis was conducted to examine the data. A password‐protected Google Sheets document was used to manage, analyze, and store data. Each team member individually reviewed raw data to achieve immersion and familiarity with the dataset and created domains to assign the data. Data analysis was conducted in three iterative coding rounds. The unit of analysis was individual utterances. Each coder completed each round independently before meeting to reach consensus prior to proceeding to the next stage. In the first round, participant utterances were simplified and rephrased while preserving their original meaning. In the second round, coders inductively generated preliminary categories and subcategories. During the third round, individual participant utterances were assigned to these categories. The conclusions were reviewed by the auditor (principal investigator) during each phase of coding to help ensure validity by verifying alignment between the raw data and the emerging analytic structure and to maintain an audit trail of coding decisions and revisions.

### 2.5 Patient and Public Involvement

The film [Blinded for Review] was created to improve community understanding of critical health topics, following recommendations from Sierra Leonean healthcare workers and mothers. This film amplifies the voices of Sierra Leonean healthcare workers and mothers, allowing for a culturally sensitive intervention that includes the perspectives of patients. Further, focus group questions were designed and edited on the basis of a practice focus group conducted with Sierra Leonean mothers.

### 2.6 Reflexivity Statement

The research team comprised six female researchers, one male researcher, and one male community partner, all co‐authors on this study: a PhD‐level principal investigator with a background in maternal and child health, a male PhD‐level principal investigator with a background in women, gender, and sexuality studies, three undergraduate students studying public health, one undergraduate student studying psychology, and one longtime male Sierra Leonean community partner who assisted with data collection, translation, and revision of the manuscript. Our diverse interdisciplinary backgrounds, along with varying levels of authority, gender identities, and lived experience in health fields, impacted underlying assumptions in our research design and interpretation of results.

## 3 Results

### 3.1 Results From Focus Groups With Mothers

FGDs with mothers (Table 1) revealed three domains: (1) perceived benefits for family well‐being, (2) health‐related outcomes, and (3) improved sense of control and advocacy.

**TABLE 1:** Results of focus groups with mothers.

Domains	Subcategories	Quotes from transcripts
Benefits to family well‐being	Family planning improves quality of life for the entire family	*With family planning my first child is being able to take care of the younger one*
	Family planning improves quality of life for children	*It [family planning] help in child nutrition because of the spacing of children, you will be able to take good care of your child*
	Family planning reduces poverty	*Family planning methods reduces poverty. Having lots of children makes no sense. It's a burden and leads to poverty*
Health benefits of family planning	Family planning prevents unwanted pregnancies	*With family planning I think I will not have too many unwanted children*
	Family planning prevents diseases	*Family planning makes you feel good and it prevent you from HIV, SYPHILIS*
Improved self‐advocacy and control	Family planning helps control the timing of pregnancy	*For me, family planning help you to plan as to what time you want a baby*
	Family planning helps mothers pursue education and other personal goals	*Family planning helps you to achieve your dreams and for school going mothers to be able to complete their educational career*
	Family planning improves confidence and preparedness	*She feels good joining family planning because she has one, after some years because of that spacing, she will have again the preparedness to have another one. The spacing is what makes her to feel good*
Stigma or negative experiences with family planning	*It has happened once when the condom got stuck in her so now she doesn't use it. She uses other methods*	

Across focus groups, mothers consistently described family planning in relation to its effects on household stability and child well‐being. The most prominent theme was improved child care, particularly through child spacing, which participants associated with better nutrition, increased parental attention, and healthier child development. Family planning was also described as enabling couples to achieve desired family size and manage caregiving responsibilities more effectively; for example, one participant noted: *“And it also helps you to take good care of your children.”* In some accounts, mothers described how they were better able to care for each child when births were spaced, noting the supportive roles of older children in caring for younger siblings. Mothers in two focus groups also frequently described family planning as a strategy for reducing poverty by limiting the number of dependents:

“For me, family planning helps to reduce poverty in one's family. Honestly, I wanted to give birth to ten children. With the help of family planning and the way things are going, I have now decided to have either one or two children only.”

The second domain focused on health‐related outcomes. The most frequently mentioned benefit in all focus groups was the prevention of unwanted pregnancies, with participants emphasizing reduced frequency of childbirth and greater control over fertility. Mothers in two focus groups explicitly linked family planning to disease prevention, including sexually transmitted infections, such as HIV and syphilis, through condom use.

The third domain centered on family planning as a tool for structuring pregnancy timing, educational attainment, and long‐term life planning. Across focus groups, mothers consistently described family planning as enabling them to determine when to have children. Participants emphasized the importance of being able to decide the timing of childbirth and ensure adequate spacing between children in order to better manage caregiving responsibilities. Three mothers also linked family planning to education attainment by enabling women to complete their education without interruption from early or unplanned pregnancies:

“Family planning helps you to achieve your dreams and for school‐going mothers to be able to complete their educational career.”

Several mothers also emphasized emotional and practical readiness associated with planned pregnancies, reporting feeling prepared when pregnancies were spaced appropriately. Some accounts in one focus group reflected changes in understanding over time, with participants describing prior misconceptions about contraceptive methods that were later addressed through education and experience, such as

“Family planning has once saved [my] life [because] during [my] youthful age [I] made the biggest mistake in [my] life because [I] didn't have education on family planning preventive measures. They said that if you engage with contraceptives you put on weight so [I] was confused. However, with lots of education and lessons learned [I am] now on the right path.”

### 3.2 Results From Focus Groups With Fathers

Fathers echoed many of the themes identified among mothers, particularly the benefits of family planning for household well‐being and maternal and child health. However, their discussions also included criticism of contraceptive methods and concerns about their reproductive consequences, resulting in a broader set of thematic domains. Specifically, FGDs with fathers (Table 2) revealed five domains: (1) criticisms and negative experiences with family‐planning methods, (2) perceived benefits for family well‐being, (3) effects on young girls, (4) health‐related benefits, and (5) the importance of collaboration in family‐planning decision‐making.

**TABLE 2:** Results of focus groups with fathers.

Domains	Subcategories	Quotes from transcripts
Criticisms and negative experience with family planning	Criticisms about health impacts	*Some preventive methods normally give our wives, friends, and our sisters frequent and excessive bleeding which normally cost them their lives*
	Concerns about infidelity and misunderstanding	*I don't like pills because it will prevent a woman from having diseases after making love with a man*
	Criticisms toward women's bodies	*Prevention increases my wife's body size and so if we want to have a child/baby, unless we go to the hospital for clean up*
	Concerns about future pregnancies	*[…] if you want to have a child at a particular time, you will not have it because of the prevention that is in your system. Even if they stop using it, it will still take them months before one could conceive through God's blessings*
	Concerns about effectiveness of family‐planning measures	*If you send someone to give you one, ask the man to use a condom, the condom may be pierced, there will be nothing to protect. You may get the STI*
Benefits to family well‐being	Family planning prevents unwanted pregnancies	*Also, family planning help to protect our wives and prevent them from unwanted pregnancy*
	Family planning reduces poverty	*With family planning it will help us to boost our financial strength and help us to take good care of our family members*
	Family planning improves maternal and child health	*Family planning helps to prevent infant mortality*
Effects on young girls	Concerns about exposing young girls to negative influences	*It will take their minds off the schooling system*
	Family planning impacts young girls’ future pregnancies	*In the long run if these girls want to give birth it will be very difficult with FP*
	Family planning helps young girls thrive	*Family planning helps to boost the medical sector and also helps in preventing our young girls from becoming pregnant at an early age*
Health benefits of family planning	*Family planning methods help in reducing the rampant sexually transmitted diseases because condoms help to protect any kind of sexually transmitted diseases*	
Importance of collaboration in family planning	*With family planning I will be able to plan my family with my wife*	

The most frequently cited concerns involved perceived negative health effects on women, including excessive bleeding, stomach pain, menstrual disruption, and general deterioration in health. Related to this, two fathers in different focus groups described noticeable changes in women's bodies, particularly increased body size, as a consequence of contraceptive use. A second set of concerns focused on future fertility, with participants in one focus group expressing fears that family planning could delay or complicate conception even after stopping these measures:

“Most times the ‘captain ban’ has been disturbing. Some women [do not have] their correct menstrual cycle or maybe disturb their child birth (delay in having pregnancy).”

In addition, fathers in three focus groups raised concerns about trust and communication within relationships, particularly voicing the anxiety that contraceptive use without full partner awareness could lead to suspicion between spouses. Finally, fathers in one focus group expressed doubts on the effectiveness of certain methods, especially condoms, which were described as potentially unreliable in preventing pregnancy or sexually transmitted infections:

“Most condoms have leakages. You get the pregnancy you're running away from.”

Fathers across all focus groups shared similar understandings about the benefits of family planning to family well‐being, particularly household stability, economic well‐being, and maternal and child health. The most frequently discussed benefit was the prevention of unwanted pregnancies. Participants noted that family planning allows families to determine the number of children they wish to have. This was closely linked to higher financial stability, with fathers emphasizing that fewer and better planned births improve their ability to provide for family members. Participants also highlighted that family planning supports child development and reduces risks of children “going wayward” [likely referring to engaging in socially undesirable or risky behaviors]. In addition, fathers emphasized reduced risk of maternal mortality during childbirth and lower rates of infant mortality. Some participants also connected family planning to broader community well‐being:

“Family planning helps us to have a healthier and sustainable environment. Family planning will increase standard of living among family members. It will also prevent the community from having disease because they are well planned and they take good care of their children and their environment.”

Beyond health and economic outcomes, family planning was also described as contributing to household harmony, with some fathers noting that reduced uncertainty around pregnancy can improve trust:

“ […] if you properly space, your children will have respect for one another.”

A prominent theme, especially in one particular focus group, was fathers’ concern for young girls. The most frequently discussed criticism is that family planning could lead young girls toward negative social influences. Fathers mentioned how contraceptive access or education could encourage early sexual activity, disobedience to parents, and distract girls from schooling. Some fathers linked family planning to “stubbornness” and vulnerability to financial influences that could lead girls into harmful situations, including prostitution. One father discussed the following:

The FP in the film, [I] disagree with it because it sends young girls to the streets, it makes them not listen to parents when there's a boy in the same community that give the girls incentives and the girl will cave in so easily.

Fathers also voiced criticism of family planning focused on future fertility. Fathers were concerned that contraceptive use at a young age could interfere with reproductive health later in life, making it more difficult for girls to conceive when they choose to start families. In contrast, very few fathers indicated that family planning could help young girls thrive by preventing early pregnancy and supporting broader health outcomes.

Fathers in one focus group briefly described health‐related benefits of family planning and the importance of collaboration. They emphasized the use of condoms as a protective method against sexually transmitted infections. Additionally, only two fathers mentioned that family planning is a collaborative process within marriage.

## 4 Discussion

This study's findings revealed significant differences in mothers’ and fathers’ attitudes about family planning, highlighting both gender‐specific concerns and broader implications for family‐planning education. A central tension that emerged among fathers was their desire to be involved in family‐planning decisions while simultaneously and consistently framing reproductive health as primarily a “woman's domain.” This contradiction reflected broader structural gender norms that both encourage male authority in household decision‐making, while also distancing men from direct responsibility for reproductive health. These findings illustrated how family‐planning decisions are shaped by women's ability to navigate and negotiate their preferences within relationships and social structures, a process we conceptualized above as interagency.

The majority of fathers mentioned negative effects on their partners’ health as a significant deterrent to using prevention methods. However, their framing of these concerns, such as complaints about “body size” and “unusual menses,” often reflected a focus on personal inconvenience rather than consideration of their partners’ needs, preferences, or well‐being. This perspective underscored persistent patriarchal norms that prioritize men's preferences and sexual desires over women's autonomy in reproductive health decision‐making, aligning with several studies on male involvement in family planning across sub‐Saharan Africa [7, 8]. When these preferences influence whether or how women can use contraception, they limit women's ability to exercise interagency within their relationships. Furthermore, some fathers expressed moral opposition to family‐planning use among adolescent girls, linking it to sexual promiscuity, prostitution, and sexually transmitted infections. This moral framing is evident in existing literature and may be better understood as patriarchal misattribution of blame and social control over female sexuality. Such perspectives reinforce gendered stigma around adolescent sexuality, ultimately limiting open dialogue about reproductive health and reinforcing structural barriers to care [11, 12]. These dynamics not only constrain the range of reproductive choices available to women and girls but also stigmatize those choices and ultimately hinder reproductive health outcomes [13, 14].

The most important finding of this study was the apparent contradiction in fathers’ perspectives on family planning. Fathers consistently described reproductive health as primarily a woman's responsibility yet simultaneously expressed a strong desire to be involved in family‐planning decisions. For some, involvement was framed as open communication and shared decision‐making between partners. For others, however, involvement appeared to center on maintaining authority over women's reproductive choices, particularly through opposition to confidential family‐planning services. This distinction is important because male involvement is frequently promoted within family‐planning programs as positive and often a strategy for improving contraceptive uptake and couple communication [15]. However, our findings suggest that involvement itself is not inherently empowering. Depending on how it is understood and enacted, greater male involvement may either support women's interagency through collaborative decision‐making or reinforce existing power imbalances by increasing men's oversight of women's reproductive choices. A qualitative study with 30 Zambian women revealed that partner involvement may also manifest as control over contraceptive decision‐making, thereby limiting women's reproductive autonomy [16]. Therefore, promoting women's interagency is not simply increasing men's participation in family planning but fostering forms of involvement that strengthen women's capacity to negotiate reproductive decisions within their relationships. Interventions should move beyond encouraging male involvement alone and distinguish between supportive partnership and controlling participation. Additionally, although fathers expressed a desire for greater involvement, mothers were not explicitly asked how they perceived men's participation in family‐planning decisions. Future research should explore how women perceive supportive and controlling partner involvement and how they practice interagency within each context.

As opposed to fathers, mothers in this study highlighted the primary benefits of family planning as increasing the health of their children and the stability of their household. Mothers’ emphasis on family planning as a means to improve their children's health and household stability underscored their role as primary caregivers. Although women's perspectives are undoubtedly shaped by prevailing gender norms, their emphasis on family well‐being should not be viewed solely as conformity to traditional caregiving roles. Rather, it illustrates how these mothers exercise interagency by navigating reproductive decisions in relation to their children's needs, household resources, educational goals, and desired family size. This positive framing may also reflect pragmatic accommodation to the gendered expectations and constraints surrounding motherhood and family life, rather than necessarily representing a challenge to existing gender norms. Advancing the well‐being of their families and pursuing their own reproductive goals are often complementary rather than competing. At the same time, several mothers described how family planning can promote more open negotiation of gendered power dynamics within domestic relationships and reduce unwanted pregnancies. Importantly, many mothers discussed educational continuity as a main benefit of family planning. Similarly, past research has linked access to family planning with women's improved control over their reproductive choices, enabling broader societal benefits like improved education, economic participation, and health outcomes [17]. For example, family‐planning programs that integrate gender equality principles can combat restrictive norms by engaging men and promoting shared responsibilities [17]. Although these changes do not reflect full reproductive autonomy, they do indicate meaningful, though constrained, improvements in women's interagency within male‐dominated household structures.

Because family planning is fundamentally a household‐level issue, effective interventions must engage both partners and the broader social environment in which reproductive decisions are made. However, in Sierra Leone, family planning remains highly stigmatized, making open discussion within families and in community settings difficult. Narrative documentaries may facilitate such discussions. As suggested by above findings and similar literature, education through personal voices and stories has been effective in Sierra Leone, engaging participants and eliciting nuanced responses about the health of the family [18]. When followed by thoughtful follow‐up questions (see Appendix 1), viewers have the opportunity to voice their opinions and discuss ideas with others. Such discussions were evident in the focus groups, as both fathers and mothers expressed concerns about stigma associated with family planning, suggesting that misinformation and social judgment remain deeply embedded across genders. When met with reassurance from healthcare workers or community members, such stigma may be resolved or reduced. Ongoing filmic interventions and intentional discussions may serve as a useful approach for promoting dialogue around family planning. A previous study on the same film *[Blinded for Review]* revealed a 61.1% increase in willingness to seek family‐planning services among mothers and a 53.4% increase in willingness to be involved in their partners’ healthcare [19]. Future studies should evaluate whether this approach leads to measurable behavior changes. Furthermore, a strong correlation exists between gender equality and improved access to contraception. When women have enhanced educational and economic opportunities, their abilities to address family‐planning needs increase significantly [20].

Taken together, these findings advanced understanding of interagency in family planning by showing that women's reproductive decision‐making is a process negotiated within intimate relationships and shaped by partner involvement, gender norms, and access to information. The findings further demonstrated that male involvement can either facilitate or constrain interagency depending on whether it supports collaborative decision‐making or reinforces control over women's reproductive choices. Thus, strengthening women's interagency requires not only improving access to family planning, but also creating relational and social conditions that support women's ability to negotiate reproductive decisions.

### 4.1 Limitations

A primary limitation of this study is the comparability of the mothers’ and fathers’ focus groups. In addition to being facilitated by two male moderators, mothers and fathers were recruited from different community settings, which may have influenced group dynamics and the topics participants chose to emphasize. These differences in recruitment context and facilitation may have affected the depth and nature of participants’ responses, limiting direct comparisons between the two groups. Another limitation of the study is the gender of the facilitator. In general, fathers provided more detailed responses to focus group questions than mothers, which may reflect participants’ comfort discussing family planning with male facilitators as well as social desirability bias. On the other hand, mothers’ responses were shorter and less detailed because of the sensitivity of the topic of family planning. The difference in quality and length of the answers compromised the comparability between two groups, which researchers have noted during the discussion. Nevertheless, the findings surrounding mothers’ more positive perceptions of family planning fit with the findings of other relevant literature.

This study has other potential limitations. The nonrepresentative, convenience sampling technique of the study limits generalizability. No critical sociodemographic data beyond rural/urban classification were collected to ensure confidentiality of participants. The absence of sociodemographic characteristics limited the transferability of our findings. Moreover, the study is subject to social desirability bias, as participants may have provided answers they thought aligned with the facilitator's knowledge, which was exacerbated by the gender of the facilitator.

### 4.2 Future Directions

This study's findings have significant implications for family‐planning education. Educational interventions need to address misinformation about side effects of family‐planning measures, especially birth control pills, more directly. Although men have had abundant resources for family planning, tailoring educational materials to address their particular concerns, including accurate information about the side effects and social implications of contraceptive use, may enhance their receptivity and support for family‐planning methods. However, these programs should not reinforce male control over reproductive decisions or shift decision‐making authority away from women. Proven strategies for men in similar contexts include outreach with influential male figures, promoting community dialogue, emerging hotlines, and engaging religious leaders [21].

The timing of family‐planning education should also be taken into account. In this study, fathers highlighted negative perceptions of exposing adolescent girls to family‐planning education, criticizing such efforts as the promotion of sexual behavior. Earlier and further inclusion of adolescents in family‐planning education, especially in schools, may reduce misinformation and perceived negative side effects of contraceptive methods. Increasing early awareness may promote a new generation of mothers and fathers with more positive perceptions of family‐planning practices. These findings contribute to the literature by highlighting the importance of addressing gender‐specific concerns and fostering open communication between partners about family planning. Our conclusions suggest how health interventions could benefit from personal stories and direct engagement that complement educational materials. Future research should explore how different approaches to education impact perceptions and behaviors related to family planning. In particular, a pilot randomized controlled trial could evaluate the efficacy of a film intervention against standard family‐planning education by assigning eligible participants or couples to receive the film intervention or a standard education. The study could assess changes in couple communication, knowledge and perceptions of contraceptive methods, willingness to use or support family planning, and subsequent contraceptive uptake. Baseline and follow‐up assessments could be used to determine whether the intervention produces changes in perceptions and communication immediately following exposure and whether these changes translate into sustained behavioral outcomes. This design would provide a more rigorous assessment of whether the film can move beyond increasing awareness to influence family‐planning behaviors.

## Author Contributions

Fathima Wakeel, Michael Kramp, and Bea Arnfield designed the study and led the submission of the grant application. Fathima Wakeel, Michael Kramp, and Bea Arnfield contributed to the design of the study, development of data collection tools, planning of the fieldwork, and coordination of the study. Bea Arnfield led the data collectors’ training. Musa Santigie Kamara participated in study design and data collection procedures. Bea Arnfield, Sophie Ritzler, and M. J. Le Vu drafted the initial version of the manuscript. All authors critically reviewed, provided feedback, and approved the final manuscript. M. J. Le Vu is responsible for the overall content as guarantor.

## Funding

This research was funded by Lehigh University College of Arts and Sciences, Lehigh University College of Health, Lehigh University Office of Creative Inquiry, and Davis Projects for Peace.

## Ethics Statement

The Lehigh University Institutional Review Board and Sierra Leone Ethics and Scientific Review Committee approved the completion of this study. Data do not contain sensitive or personal information. This research adheres to all ethical policies of Wiley: Public Health Challenges.

## Consent

All participants gave informed consent.

## Conflicts of Interest

The authors declare no conflicts of interest.

## Supporting information




**Supporting file 1:** puh270430‐sup‐0001‐SuppMat.docx

## Data Availability

The data that support the findings of this study are available on request from the corresponding author. The data are not publicly available due to privacy or ethical restrictions.
