# Supplementary material for: The Challenge of Sexual Reproductive Health in Sierra Leone: Gendered Perceptions of Family Planning
Source: Public Health Chall. 2026 Nov 6;5(4):e70430. doi: 10.1002/puh2.70430 (PMC13742369; doi:10.1002/puh2.70430)
Supplement: Supplementary file 1 [file PUH2-5-e70430-s001.docx]

**Appendix 1**

**Focus Group Questions**

For Mothers:

*QUESTION ONE (1): WHAT DID YOU LEARN FROM THE FILM?*

*QUESTION TWO (2): WHAT DID YOU LIKE ABOUT THE FILM?*

*QUESTION THREE (3): WHAT DID YOU DISLIKE ABOUT THE FILM?*

*QUESTION FOUR (04): HOW HAS THE FILM CHANGED YOUR ATTITUDE ABOUT SEEKING HEALTHCARE FOR YOU AND YOUR CHILD?*

*QUESTION FIVE (05): HOW IMPORTANT IS FAMILY PLANNING?*

*QUESTION SIX (06): DOES THE FILM REFLECT YOUR EXPERIENCE AS A MOTHER OR PATIENT IN A CLINIC? IF YES, HOW?*

*QUESTION SEVEN (07): WHAT ROLE DOES YOUR HUSBAND/PARTNER HAVE IN YOUR HEALTHCARE OR YOUR CHILD’S HEALTHCARE?*

For Fathers:

*QUESTION ONE: WHAT DID YOU LEARN FROM THE FILM?*

*QUESTION TWO: WHAT DID YOU LIKE ABOUT THE FILM?*

*QUESTION THREE: WHAT DID YOU DISLIKE ABOUT THE FILM?*

*QUESTION FOUR: HOW IMPORTANT IS FAMILY PLANNING?*

*QUESTION FIVE: DOES THE HEALTHCARE SYSTEM INCLUDE FATHERS IN FAMILY PLANNING DECISIONS?*

*QUESTION SIX: WHAT ARE YOUR GREATEST CONCERNS ABOUT THE FAMILY PLANNING METHODS YOUR WIFE RECEIVE?*

*QUESTION SEVEN: DID THE FILM ENCOURAGED YOU TO BE MORE INVOLVED IN YOUR WIFE’S FAMILY PLANNING DECISIONS?*

*QUESTION EIGHT: AS A FATHER, WHAT DO YOU SEE AS YOUR ROLE IN HELPING YOUR WIFE OR PARTNER GET THE HEALTHCARE SERVICES SHE NEEDS?*
